# Trends in stroke subtypes and vascular risk factors in a stroke center in China over 10 years

**DOI:** 10.1038/s41598-018-23356-9

**Published:** 2018-03-22

**Authors:** Danyang Tian, Qiong Yang, Quan Dong, Nan Li, Bernard Yan, Dongsheng Fan

**Affiliations:** 1 Department of Neurology, Peking University Third Hospital, Beijing, China; 2 Research Center of Clinical Epidemiology, Peking University Third Hospital, Beijing, China; 3 Department of Medicine and Neurology, Melbourne Brain Centre at the Royal Melbourne Hospital, University of Melbourne, Parkville, Australia; 4 Key Laboratory for Neuroscience, Ministry of Education/National Health & Family Planning Commission, Peking University, Beijing, China

## Abstract

Rapid economic development in China has caused marked changes in people’s lifestyles and medical technology. Exploration of stroke subtype trends is necessary to provide physicians with vital insight for early diagnosis and treatment. We included stroke patients admitted from 2006 to 2015. Trends in stroke subtypes and vascular risk factors were investigated. There were 5521 patients, including 4534 (82.1%) ischemic stroke (IS), 813 (14.7%) intracerebral hemorrhage (ICH) and 174 (3.2%) subarachnoid hemorrhage (SAH) patients. The proportion of IS was increasing and proportions of ICH and SAH were decreasing (P < 0.001). Onset age and hypertension remained stable in stroke subtypes. In IS patients, large artery atherosclerosis (LAA) strokes increased from 17.0% to 30.8% in the first 7 years and ultimately decreased to 24.1%. Small vessel disease (SVD) strokes increased from 15.5% to 39.6%, undetermined etiology (UE) strokes decreased from 52.7% to 26.0%, others remained stable. The levels of low-density lipoprotein declined significantly, and an increased number of patients underwent intracranial artery examinations (P < 0.001). In conclusion, proportions of stroke subtypes changed significantly. Anti-hypertension therapy needs to be reinforced to control ICH, SAH and SVD ischemic stroke incidences. The etiologic detection of IS increased and lipid-lowing therapy was effective, cardioembolism detections should be reinforced.

## Introduction

Strokes are highly prevalent and cause major disabilities worldwide. Both ischemic stroke (IS) and hemorrhage stroke could cause high mortality^1^. Changes in lifestyle and advances in medical technology during the past decade have likely affected the prevalence of vascular risk factors and thereby the incidence of stroke. It is important for clinicians to have a general impression of the distribution of stroke subtypes to adjust strategies for etiologic detections and to make decisions in diagnosis and therapy^2^.

IS has been classified into 5 etiologic subtypes, which differ in terms of mechanism, severity, management and prognosis^3^. Over the past decade, 2 major factors should be considered. First, statins are reported to lower stroke incidence and recurrence through multiple mechanisms^4,5^. Guidelines for secondary and primary prevention of strokes have recommended the use of statins successively, which would likely lead to enhanced anti-atherosclerosis therapy and a decreased incidence of large artery atherosclerosis (LAA) stroke cases^6^. Second, the aging of the population might influence some age-related risk factors, such as atrial fibrillation (AF) and, consequently, the number of cardioembolic (CE) stroke cases^7^.

A few studies have reported IS subtype trends in several other countries^2,8–10^. However, these findings have been inconsistent, and no data from China have been reported. China has different social conditions from other countries and has experienced vast economic development. Therefore, we intended to explore the trends in stroke subtypes, etiology of IS and risk factors in a stroke center in China.

## Methods

### Study setting and sample

This was a retrospective, hospital-based study. Patients with acute ischemic or hemorrhagic stroke (defined as ≤14 d) admitted to Peking University Third Hospital from January 2006 to December 2015 were included. We excluded patients less than 18 years old and recurrent IS patients in our hospital because the latter cases were likely due to the same IS etiology and may have resulted in an overestimation of the distribution. The patients mainly resided in the Beijing District, the capital city of China. The research was approved by the ethics committee of Peking University Third Hospital. Informed consent was obtained. All methods were performed in accordance with the relevant guidelines and regulations.

The collected data included demographics, stroke risk factors, etiology investigations and ischemic stroke subtypes. Blood pressure was measured on admission. Laboratory investigations were conducted in a fasting state on the second morning after admission. We combined etiologic detection findings by different image technologies to calculate the accomplishment rate in IS patients.

Ischemic subtypes were classified according to the Trial of ORG 10172 in Acute Stroke Treatment (TOAST) criteria^11^. Briefly, stroke patients were divided into 5 subtypes: LAA, CE, small vessel disease (SVD), other determined etiology (ODE) and undetermined etiology (UE). Specifically, patients with a ≤2 cm size infarct and >50% relevant large artery stenosis on neuroimaging were categorized as having LAA. Ischemic stroke subtypes were evaluated by two independent physicians, and the uncertain cases were discussed to reach a consensus.

### Statistical analysis

Quantitative data are presented as the means ± standard deviations (SDs), and qualitative data are presented as numbers and percentages. The data were divided into 3 periods according to admission date: from January 2006 to December 2008, from January 2009 to December 2012, and from January 2013 to December 2015. Analysis of variance (ANOVA) and Chi-squared tests were used for continuous and discrete data separately to analyze the changes in the risk factors among the 3 periods. Binary logistic regression was used to analyze the change in each IS subtype. In each subtype, the proportion in the subsequent year and the average proportions in the earlier years were compared to determine whether significant changes occurred over time. The trends of LAA and SVD strokes were further adjusted for the using vascular investigations. The trend of CE stroke was further adjusted for the using Holter monitoring and echocardiography. All P values are two-sided, and the probability of a type 1 error was set as 0.05. Statistical analyses were performed using SPSS 17.0.

## Results

A total of 6483 patients with stroke were admitted to Peking University Third Hospital; 359 patients were excluded because they did not meet the criteria for acute phase stroke, which is less than 14 days after an ischemic stroke. Additionally, 603 patients who visited the same hospital for the second time due to a recurrent stroke were excluded. Therefore, 5521 patients were enrolled in our study. There were 4534 (82.1%) IS patients, 813 (14.7%) intracerebral hemorrhage (ICH) patients and 174 (3.2%) subarachnoid hemorrhage (SAH) patients, 3888 (70.4%) were males. The mean ages were 63.51 ± 13.43 years. In IS patients, there were 3256 (71.8%) males and the mean ages were 64.53 ± 12.82 years. In ICH patients, there were 551 (67.8%) males and the mean ages were 59.33 ± 15.28 years. In SAH patients, there were 81 (46.6%) males and the mean ages were 56.47 ± 13.84 years.

Demographic data and risk factors among the 3 periods are shown in Table 1. Totally, the proportion of IS was increasing and the proportions of ICH and SAH were decreasing (P < 0.001). Onset age did not show a significant change over time (P = 0.337 in IS patients, 0.370 in ICH patients, 0.490 in SAH patients). Male was significantly increasing in IS patients but not in ICH or SAH patients (P = 0.001, 0.695 and 0.596 separately). The mean length of hospital stay continually decreased from 18 days to 14.8 days in IS patients(P < 0.001), and from 19.2 days to 16.4 days in ICH patients (P = 0.014), but did not change significantly in SAH patients (P = 0.302). Regarding the risk factors, the proportion of hypertension remained stable (P = 0.995 in IS patients, 0.927 in ICH patients and 0.453 in SAH patients), and the proportion of diabetes mellitus increased from 29.8% to 40.1% in IS patients (P < 0.001).

**Table 1:** Demographics, vascular risk factors and investigations among the 3 periods in all patients.

	2006–2008 (n = 1410)	2009–2012 (n = 2209)	2013–2015 (n = 1728)	P value
**Subtypes, n (%)**				<0.001
IS	1124(75.4%)^†^	1874(82.4%)	1536(87.5%)	
ICH	286(19.2%)^†^	335(14.7%)	192(10.9%)	
SAH	80(5.4%)^†^	66(2.9%)	28(1.6%)	
**Demographic factors**				
Age, y, Mean ± SD				
Age in IS	64.97 ± 12.43	64.26 ± 13.06	64.53 ± 12.80	0.337
Age in ICH	59.62 ± 14.88	59.86 ± 15.60	57.98 ± 15.29	0.370
Age in SAH	55.35 ± 13.64	56.77 ± 14.82	58.93 ± 12.02	0.490
Males, n (%)				
Males in IS	760(67.6%)^†^	1362(72.7%)	1134(73.8%)	0.001
Males in ICH	195(68.2%)	222(66.3%)	134(69.8%)	0.695
Males in SAH	38(47.5%)	28(42.4%)	15(53.6%)	0.596
**Duration, d, Mean ± SD**				
Duration in IS	18.01 ± 8.45^†^	16.57 ± 7.99^‡^	14.81 ± 7.43	<0.001
Duration in ICH	19.22 ± 9.80	18.43 ± 11.62^‡^	16.38 ± 9.71	0.014
Duration in SAH	18.73 ± 10.77	19.50 ± 13.65	15.50 ± 7.59	0.302
**Risk factors, n (%)**				
Smoking				
Smoking in IS	449(39.9%)	695(37.1%)	583(38.0%)	<0.001
Smoking in ICH	69(24.1%)	68(20.3%)	37(19.3%)	0.408
Smoking in SAH	17(21.3%)	7(10.6%)	4(14.3%)	0. 091
**Drinking**				
Drinking in IS	362(32.2%)	575(30.7%)	474(30.9%)	<0.001
Drinking in ICH	76(26.6%)	72(21.5%)	39(20.3%)	0.256
Drinking in SAH	15(18.8%)	8(12.1%)	3(10.7%)	0.174
**Hypertension**				
Hypertension in IS	806(71.7%)	1341(71.6%)	1099(71.5%)	0.995
Hypertension in ICH	207(72.4%)	247(73.7%)	141(73.4%)	0.927
Hypertension in SAH	5(6.3%)	8(12.1%)	3(10.7%)	0.453
**Diabetes mellitus**				
Diabetes mellitus in IS	335(29.8%) ^†^	673(35.9%)	616 (40.1%)	<0.001
Diabetes mellitus in ICH	47(16.4%)	65(19.4%)	33(17.2%)	0.606
Atrial fibrillation ^§^	83(7.4%)	145(7.7%)	110(7.2%)	0.812
CHD ^§^	121(10.8%)	279 (14.9%)	172(11.2%)	0.001
Ischemic stroke ^§^	12(1.1%)^†^	90(4.8%)^‡^	121(7.9%)	<0.001
**Assays, Mean ± SD**				
SBP (mmHg) in IS	148.0 ± 22.3^†^	144.8 ± 21.0	144.2 ± 20.8	<0.001
SBP (mmHg) in ICH	156.0 ± 27.8	152.3 ± 27.4	150.3 ± 27.9	0.073
SBP (mmHg) in SAH	133.1 ± 21.3^†^	140.6 ± 21.6	131.2 ± 20.7	0.060
DBP (mmHg) in IS	85.1 ± 12.4^†^	83.9 ± 12.2^‡^	81.9 ± 12.4	<0.001
DBP (mmHg) in ICH	89.5 ± 16.6	88.0 ± 16.4	86.7 ± 14.9	0.156
DBP (mmHg) in SAH	78.1 ± 12.7	80.4 ± 12.2	75.4 ± 10.8	0.194
TC (mmol/L)^§^	4.97 ± 0.94^†^	4.52 ± 1.02^‡^	4.12 ± 1.01	<0.001
LDL-C (mmol/L)^§^	3.16 ± 0.86^†^	2.77 ± 0.82^‡^	2.50 ± 0.84	<0.001
HDL-C (mmol/L)^§^	1.15 ± 0.40^†^	0.98 ± 0.24	0.98 ± 0.23	<0.001
TG (mmol/L)^§^	1.90 ± 1.17^†^	1.75 ± 1.05^‡^	1.67 ± 1.12	<0.001
Glucose (mmol/L)^§^	6.13 ± 2.46	6.10 ± 2.43	6.12 ± 2.27	0.906
HCY (mmol/L)^§^	20.05 ± 29.84^†^	18.38 ± 12.95^‡^	16.41 ± 10.24	<0.001
**Investigations** ^**§**^ **, n (%)**				
Intracranial artery*	288(25.6%)^†^	1145(61.1%)^‡^	1158(75.4%)	<0.001
Extracranial artery^#^	919(82.4%)	1544(83.0%)^‡^	1349(87.8%)	<0.001
Echocardiography	528(47.4%)^†^	1293(69.5%)^‡^	1268(82.6%)	<0.001
Holter monitoring	4(0.4%)	3(0.2%)^‡^	126(8.2%)	<0.001

^*^The investigations include transcranial doppler, magnetic resonance angiography of the intracranial artery, computerized tomography angiography of the intracranial artery and digital subtraction angiography. ^#^The investigations include carotid duplex ultrasound, cervical magnetic resonance angiography, cervical computerized tomography angiography and digital subtraction angiography. ^†^P < 0.05 when the data in the period of 2006–2009 were compared with the data in the period of 2010–2012. ^‡^P < 0.05 when the data in the period of 2010–2012 were compared with the data in the period of 2013–2015. ^§^The data was only investigated in IS patients.

**Figure 1:**
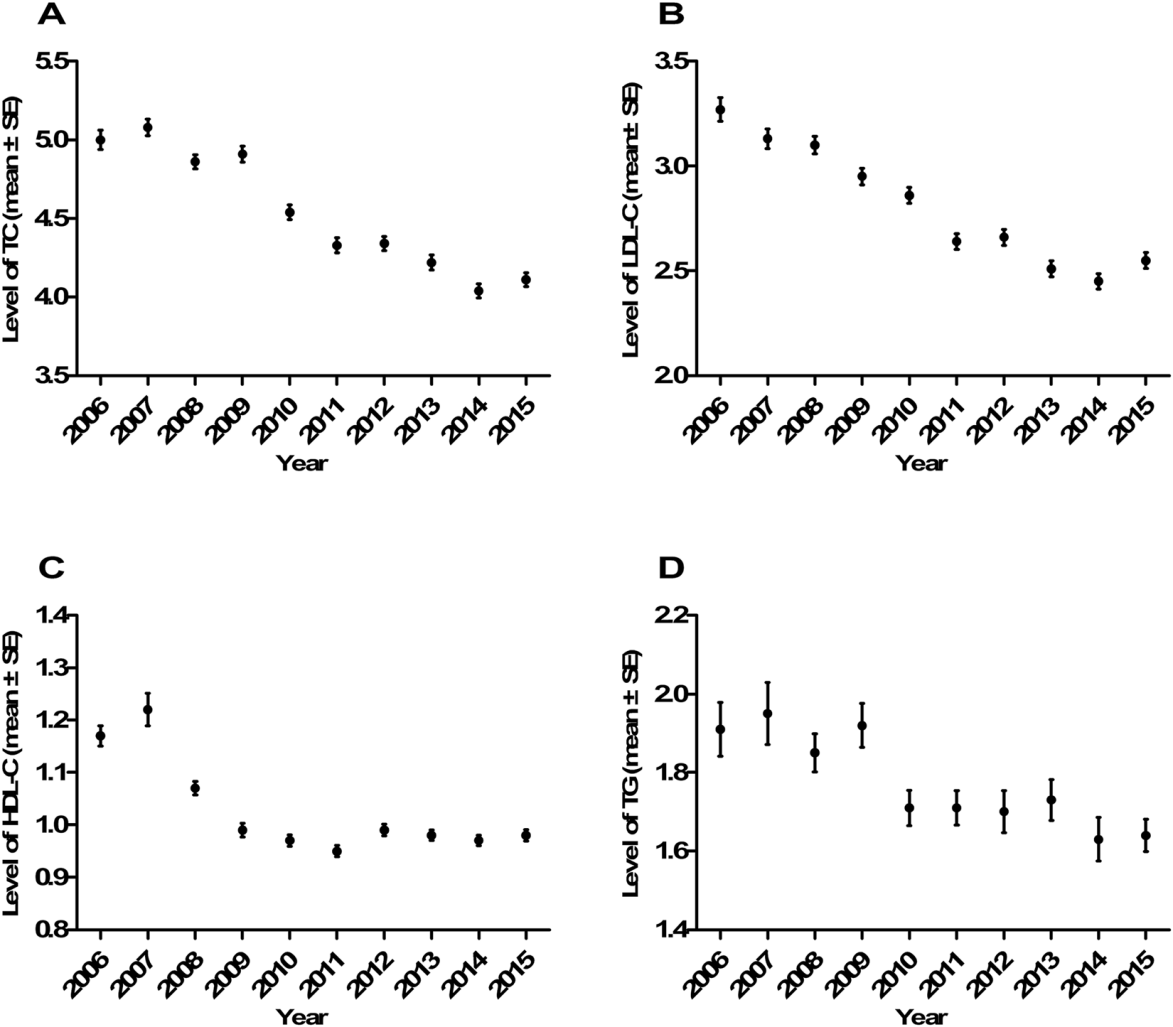
Lipid profile levels for each year in IS patients. (**A**) Total cholesterol level. (**B**) Low density lipoprotein level. (**C**) High density lipoprotein level. (**D**) Triglycerides level. SE, standard error.

**Figure 2:**
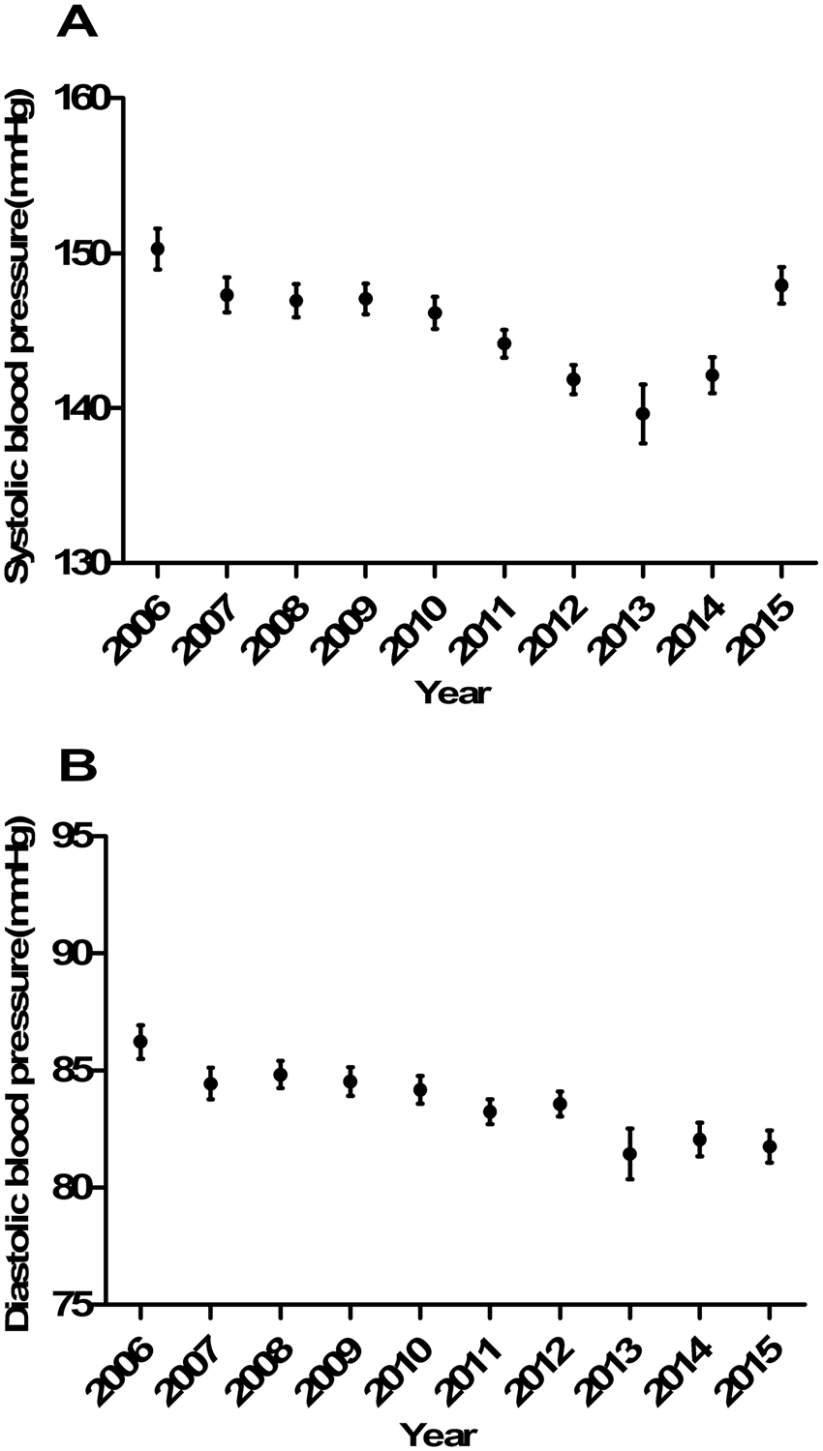
Blood pressure on admission for each year in IS patients. (**A**) Systolic blood pressure. (**B**) Diastolic blood pressure. SE, standard error.

**Figure 3:**
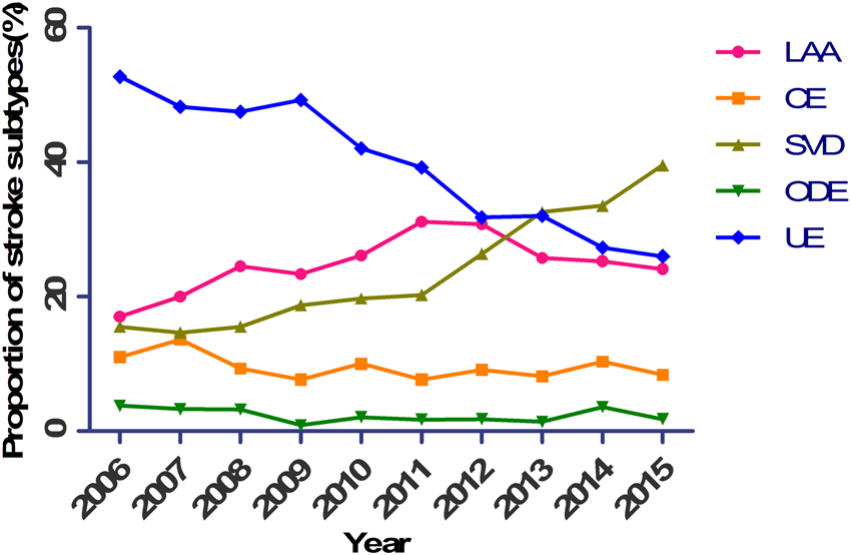
Trend of each IS stroke subtype during the past decade. LAA, large artery atherosclerosis; CE, cardioembolic; SVD, small vessel disease; ODE, other determined etiology; UE, undetermined etiology.

When we analyzed IS patients separately, we found that lipid profiles, specifically total cholesterol, triglycerides and low-density lipoprotein decreased continuously during these years (Fig. 1), systolic blood pressure decreased in the former 2 periods and diastolic blood pressure decreased in all 3 periods (Fig. 2). The proportion of AF remained stable (P = 0.812). Additionally, we observed marked increases in the use of intracranial vascular investigations, echocardiography and Holter monitoring (from 25.6% to 75.4%, from 47.4% to 82.6% and from 0.4% to 8.2%, respectively, P < 0.001). The proportion of ischemic  stroke subtypes and the association with time are shown in Tables 2 and 3 and Fig. 3.

**Table 2:** Proportions of ischemic stroke subtypes in each year.

	2006 (n = 317)	2007 (n = 360)	2008 (n = 440)	2009 (n = 428)	2010 (n = 468)	2011 (n = 469)	2012 (n = 493)	2013 (n = 512)	2014 (n = 534)	2015 (n = 439)
LAA	54(17.0%)	72(20.0%)	108(24.5%)	100(23.4%)	122(26.1%)	146(31.1%)	152(30.8%)	132(25.8%)	135(25.3%)	106(24.1%)
CE	35(11.0%)	49(13.6%)	41(9.3%)	33(7.7%)	47(10.0%)	36(7.7%)	45(9.1%)	42(8.2%)	55(10.3%)	37(8.4%)
SVD	49(15.5%)	53(14.7%)	68(15.5%)	80(18.7%)	92(19.7%)	95(20.3%)	130(26.4%)	167(32.6%)	179(33.5%)	174(39.6%)
ODE	12(3.8%)	12(3.3%)	14(3.2%)	4(0.9%)	10(2.1%)	8(1.7%)	9(1.8%)	7(1.4%)	19(3.6%)	8(1.8%)
UE	167(52.7%)	174(48.3%)	209(47.5%)	211(49.3%)	197(42.1%)	184(39.2%)	157(31.8%)	164(32.0%)	146(27.3%)	114(26.0%)

LAA, large artery atherosclerosis; CE, cardioembolic; SVD, small vessel disease; ODE, other determined etiology; UE, undetermined etiology.

**Table 3:** Trends in ischemic stroke subtypes compared in total and compared between the index year and the average level of the former years.

	P	OR (95% CI)	Adj. P*	Adj. OR (95% CI)
LAA subtype				
total	0.000		0.006	
2007 vs. 2006^†^	0.323	1.218(0.824–1.799)	0.558	1.127(0.756–1.678)
2008 vs. 2007	0.015	1.436(1.072–1.923)	0.004	1.549(1.149–2.089)
2009 vs. 2008	0.199	1.193(0.912–1.561)	0.855	1.026(0.778–1.353)
2010 vs. 2009	0.024	1.320(1.037–1.680)	0.959	1.007(0.783–1.293)
2011 vs. 2010	0.000	1.601(1.280–2.001)	0.514	1.081(0.855–1.368)
2012 vs. 2011	0.001	1.460(1.179–1.808)	0.672	1.050(0.839–1.312)
2013 vs. 2012	0.498	1.078(0.868–1.338)	0.028	0.778(0.621–0.974)
2014 vs. 2013	0.716	1.040(0.842–1.284)	0.035	0.791(0.636–0.983)
2015 vs. 2014	0.823	0.974(0.773–1.227)	0.051	0.790(0.623–1.001)
CE subtype				
total	0.142		0.020	
2007 vs. 2006	0.312	1.269(0.799–2.016)	0.338	1.255(0.789–1.996)
2008 vs. 2007	0.127	0.735(0.495–1.092)	0.149	0.745(0.499–1.111)
2009 vs. 2008	0.044	0.662(0.443–0.989)	0.037	0.651(0.435–0.974)
2010 vs. 2009	0.912	0.981(0.695–1.384)	0.774	0.950(0.672–1.343)
2011 vs. 2010	0.100	0.733(0.506–1.062)	0.037	0.672(0.462–0.977)
2012 vs. 2011	0.684	0.933(0.668–1.304)	0.271	0.825(0.586–1.162)
2013 vs. 2012	0.307	0.838(0.597–1.176)	0.133	0.769(0.546–1.083)
2014 vs. 2013	0.532	1.101(0.814–1.489)	0.843	0.969(0.711–1.321)
2015 vs. 2014	0.452	0.873(0.613–1.243)	0.067	0.698(0.475–1.025)
SVD subtype				
total	0.000		0.000	
2007 vs. 2006	0.790	0.944(0.619–1.439)	0.761	0.937(0.614–1.428)
2008 vs. 2007	0.867	1.029(0.737–1.436)	0.841	1.035(0.741–1.445)
2009 vs. 2008	0.098	1.282(0.956–1.719)	0.121	1.262(0.940–1.694)
2010 vs. 2009	0.067	1.282(0.982–1.673)	0.110	1.246(0.952–1.631)
2011 vs. 2010	0.069	1.2660(0.982–1.634)	0.150	1.212(0.933–1.576)
2012 vs. 2011	0.000	1.717(1.368–2.154)	0.000	1.655(1.311–2.088)
2013 vs. 2012	0.000	2.148(1.745–2.644)	0.000	2.073(1.674–2.567)
2014 vs. 2013	0.000	2.033(1.666–2.482)	0.000	1.974(1.610–2.420)
2015 vs. 2014	0.000	2.447(1.989–3.010)	0.000	2.394(1.942–2.952)
**ODE subtype**				
total	0.064			
2007 vs. 2006	0.751	0.876(0.388–1.980)		
2008 vs. 2007	0.739	0.892(0.456–1.744)		
2009 vs. 2008	0.012	0.266(0.094–0.750)		
2010 vs. 2009	0.673	0.857(0.419–1.754)		
2011 vs. 2010	0.364	0.703(0.328–1.506)		
2012 vs. 2011	0.538	0.799(0.390–1.634)		
2013 vs. 2012	0.227	0.615(0.279–1.353)		
2014 vs. 2013	0.037	1.739(1.035–2.920)		
2015 vs. 2014	0.602	0.823(0.395–1.714)		
**UE subtype**				
total	0.000			
2007 vs. 2006	0.259	0.840(0.621–1.137)		
2008 vs. 2007	0.326	0.887(0.697–1.128)		
2009 vs. 2008	0.942	0.992(0.793–1.240)		
2010 vs. 2009	0.005	0.743(0.603–0.916)		
2011 vs. 2010	0.001	0.700(0.570–0.860)		
2012 vs. 2011	0.000	0.538(0.438–0.661)		
2013 vs. 2012	0.000	0.593(0.485–0.724)		
2014 vs. 2013	0.000	0.505(0.413–0.618)		
2015 vs. 2014	0.000	0.508(0.407–0.635)		

^*^The adjusted factor was intracranial artery investigation in LAA-related strokes and SVD-related strokes, and the adjusted factors were echocardiography and Holter monitoring in CE-related strokes. ^†^The former year refers to the index year and the later years refers to the average level of the years before the index year.

The incidence of LAA stroke was observed to increase significantly from 17% to 30.8% from 2006 to 2013, and then decrease to 24.1% by 2015. After adjusting for the use of intracranial vascular investigations, no significant increase was observed from 2009 to 2012, and a significant decrease was observed from 2012 to 2015. An increase in SVD strokes was observed, from 15.5% to 39.6%. This trend was still significant after adjusting for the use of intracranial vascular investigations. A significant decrease in UE-related strokes was observed, from 52.7% to 26%. No significant change was observed in the proportion of CE stokes (from 11.0% to 8.4%) in the past decade, even after adjusting for using Holter monitoring and echocardiography. No significant change in ODE strokes was observed.

## Discussion

This study showed a temporal trend in stroke subtypes over the past decade, that warrants investigation considering the vast economic development in China. According to World Bank classifications, China has experienced extremely rapid economic growth and has reached a middle income level^12^. Medical resources have improved, especially in Beijing, the capital of China. Marked changes in industrial and social factors have influenced people’s lifestyle (especially diet composition) and concepts of disabling disease management, such as that for stroke. The aforementioned factors ultimately influence the stroke subtype distribution.

Generally speaking, although the proportion of IS, ICH and SAH patients have changed, they all represented a relatively stable onset age, which is different from a population-based study investigating a low-income district in Tianjin, China, the latter one showed a younger trend of onset age in the past 2 decades^13^. We suggested one of the reason was that patients in this study were located in a relatively higher income district compared with township in Tianjin, and the stroke prevention worked better. However, the risk factors correlated with specific IS subtypes was changed. The details are as below.

According to previous studies, the proportion of LAA strokes ranges from 14.4% to 57%, which is influenced by ethnicity, region, lifestyle and other factors^2,8^. In this study, the proportions of the LAA subtype ranged from 17% to 30.8% in different time periods, consistent with other studies in China^14^. Reportedly, LAA stroke patients have a higher risk of cardiovascular events, stroke recurrence and death^15^. Atherosclerosis, especially carotid plaque and carotid intima-media thickness, is also closely associated with cardiovascular risk, which is closely associated with risk factors such as blood lipid levels^16,17^. Statins reportedly aid in the prevention of stroke incidence and recurrence. Since the guidelines for primary stroke prevention recommended statins for anti-atherosclerosis therapy in 2011, statins began to be widely applied^6^.

The proportion of LAA subtype patients increased in the first 7 years, but decreased in the last 3 years. As we divided the past decade into 3 periods previously, the change was not significant in the 1^st^ and 3^rd^ periods, but was significant in the 2^nd^ period. After adjusting for intracranial vascular investigations, the increasing trend in the 2^nd^ period became non-significant, and the decreasing trend in the 3^rd^ period became significant. Notably, the use of intracranial vascular investigations began to increase in 2009, i.e., the beginning of the 2^nd^ period. Statins were not recommended until 2011, and the 3^rd^ period followed. Therefore, we presumed that the increasing trend in LAA strokes in the 2^nd^ period was due mostly to increasing detection abilities and the availability of stable anti-atherosclerosis therapy. In other words, more LAA stroke subtype patients were diagnosed. In the 3^rd^ period, the etiology detection increased slightly and anti- atherosclerosis therapy was strengthened by using statins, resulting in a significant downtrend of LAA stroke cases. This finding is also consistent with the decreasing lipid profiles and implies that a positive outcome might be achieved by anti-atherosclerosis therapy for the control of LAA strokes.

Hypertension appears to be the most predominant risk factor in the population, contributing to half of the coronary heart disease burden and approximately two-thirds of the cerebrovascular disease burden^18^. Hypertension is more likely to be correlated with the SVD subtype than the other subtypes in ischemic stroke patients^19^. Lowering blood pressure could reduce the risk of stroke. Reportedly, when the blood pressure is reduced by 8/4 mmHg, the risk of stroke and other cardiovascular events obviously decreases^20^. To clarify the underlying reason for the rising trend in the SVD stroke cases, we should first focus on the prevalence and control of hypertension. Based on previous community studies, the hypertension control rate is 9.3% in China compared with 33.1% in America and is also much lower than in several other Asian countries such as Korea, Thailand, and Iran^18,21,22^. In our study, less efficacy was also shown in hypertension control. The number of patients with hypertension remained stable over the past decade, and the decrease in blood pressure was slight. Briefly, anti-hypertensive therapy should be reinforced to control the incidence of SVD stroke cases.

Unlike those in other countries, CE stroke cases did not increase in our study. The proportion fluctuated between 11% and 8.4%, much lower than the values reported in other studies such as the German stroke data bank (25% CE strokes)^23,24^. Once CE sources are recognized, anti-coagulation treatment should start. Early diagnosis is very important.

AF is the most common cause in CE stroke cases. In this study, stroke with AF accounted for approximately 7% to 8% of all ischemic  stroke types, similar to values reported in Canada (8–11%) but much lower than values reported in Poland (24.6–31.7%). There are several risk factors for AF, such as hypertension, coronary heart disease and age^25,26^. A high proportion of coronary heart disease and the increasing onset age may contribute to the high AF rate in Poland. The onset age in our study did not increase, possibly partially due to the less effective measures for risk factors, since successful control measures for hypertension, diabetes mellitus and dyslipidemia are suspected to delay the onset age of strokes. Additionally, we also need to consider the detection rate. Paroxysmal AF becomes more detectable as the electrocardiographic (ECG) monitoring period is prolonged^27^. Holter monitoring is recommended to improve AF detection. In our study, all patients underwent 12-lead ECG monitoring on admission, but the proportion of patients who underwent 24-h Holter monitoring was only 0.4–8.2%, which was much lower than in reports from Canada (13–46%) and Poland (26–62.5%)^2,8^. It is easy to conclude that AF detection in China is less valued and needs to be reinforced among UE stroke patients.

The time trends in stroke subtypes in this study were much different from those of other countries. Here, we summarized previous studies in Canada, Korea, Poland and Japan^2,8,10,22^. An increasing trend was observed in CE strokes, and a decreasing trend was observed in SVD strokes in Canada, Korea, and Poland. However, LAA strokes declined in Canada, remained stable in Korea, and showed an increasing trend in Poland. A community-based study in Japan showed an increasing trend in CE strokes, with a decreased trend in non-lacunar strokes. Consistently, the UE stroke subtype remained stable in previous studies. In China, LAA stroke showed a single apex trend, CE stroke was low and stable, SVD stroke kept increasing and UE stroke kept decreasing, findings that are much different from those from other countries.

We also investigated the trend of ICH and SAH in the past decade. We found that there is a relatively stable proportion of hypertension, which suggests us the hypertension management should also be improved in the prevention of hemorrhagic stroke.

Our study has several advantages. Although several studies have investigated the stroke subtype trends, the situation in China remains unknown. China has different ethnic and lifestyle conventions than Western European and North American countries, and its economic status is different from other reported Asian countries, such as Korea and Japan. Considering the severe disease burden in China^28^, further investigations of the stroke subtype distribution and trends are necessary. Our study had a large sample size, which included patients admitted to our stroke center over 10 years. The electronic medical records in the hospital were accurate, and the evaluation of the TOAST subtype by neurological physicians was relatively precise.

However, this study has several limitations. First, this is a retrospective study, and some data, such as the national institutes of health stroke scale (NIHSS) score, were not available for all patients. Therefore, we cannot compare the stroke severity in different years, which may influence the proportion of stroke subtypes. Second, this is a hospital-based study. Therefore, the interpretation of the etiology should be restricted to this center, although the changes in etiology may be influenced by patients’ tendency to seek medical care and changes in the natural population. However, based on these data, the number of admitted stroke patients each year and the average age of the patients did not substantially change, suggesting a relatively steady patient source. Third, this is a single-center study and may not be fully representative of all Chinese patients. Therefore, multi-center and population-based studies are needed to determine the changes in stroke subtypes and stroke risk factors over time.
